# *Ixeris dentata* and *Lactobacillus gasseri* media protect against periodontitis through Nrf2-HO-1 signalling pathway

**DOI:** 10.1038/s41598-023-39853-5

**Published:** 2023-08-08

**Authors:** Hwa-Young Lee, Geum-Hwa Lee, Ji-Hyun Kim, Jinhua Cheng, Joo-Hyung Cho, Joo-Won Suh, Han-Jung Chae

**Affiliations:** 1 Non-Clinical Evaluation Center Biomedical Research Institute, Jeonbuk National University Hospital, Jeonju, Jeonbuk 54907 South Korea; 2 Research Institute of Clinical Medicine of Jeonbuk National University-Biomedical Research Institute of Jeonbuk National University Hospital, Jeonju, Jeonbuk 54907 Republic of Korea; 3 Myongji Bioefficacy Research Center, Myongji University, Yongin, Republic of Korea; 4 School of Pharmacy, Jeonbuk National University, Jeonju, Jeonbuk 54896 South Korea

**Keywords:** Cell biology, Molecular biology, Diseases, Health care

## Abstract

Periodontitis is an infectious inflammation in the gums characterized by loss of periodontal ligaments and alveolar bone. Its persistent inflammation could result in tooth loss and other health issues. *Ixeris dentata* (IXD) and *Lactobacillus gasseri* media (LGM) demonstrated strong antioxidant activity, which may prevent oxidative and inflammatory periodontitis. Here, IXD and LGM extracts were investigated for antioxidative activity against oral discomfort and evaluated for their synergistic effect against oxidative and inflammatory periodontitis in a mouse model. IXD/LGM suppressed pro-inflammatory cytokines like interleukin (IL)-1β, IL-6, and TNF-α. Additionally, it reduced pro-inflammatory mediators, nitric oxide, iNOS (inducible nitric oxide synthase), and COX-2 (cyclooxygenase-2) and enhanced AKT, Nrf2, and HO-1 activation. Similarly, IXD/LGM treatment elevated osteogenic proteins and mRNAs; alkaline phosphatase, collagen type 1 (COL1), osteopontin (OPN), and runt-related transcription factor 2 (RUNX2). Hematoxylin and Eosin (H&E) staining and micro-CT analysis confirm the positive impact of IXD/LGM on the periodontal structure and its associated inflammation. These findings demonstrate that IXD/LGM inhibits oxidative stress, periodontal inflammation, and its resultant alveolar bone loss in which Akt (also known as protein kinase B)-nuclear factor-erythroid 2-related factor 2 (Nrf2)-hemoxygenase-1 (HO-1) signaling is involved. Thus, IXD/LGM is a potential candidate against oxidative/inflammatory stress-associated periodontitis.

## Introduction

The chronic inflammatory response to bacterial plaques results in periodontitis, an epidemic that causes periodontal inflammation featuring tartar deposition on the teeth. This accumulation potentially leads to loss of the periodontal ligament and alveolar bone^1^. Generally, periodontitis upregulates pro-inflammatory cytokines, which stimulate inflammatory cells and destroy the alveolar bone^2^. Inducible nitric oxide synthase (iNOS) and cyclooxygenase-2 (COX-2) induced nitric oxide (NO) activates several molecular patterns along with pro-inflammatory cytokines like tumor necrosis factor-α (TNF-α), interleukin (IL)-6, and IL-1β^3^. These cytokines contribute significantly to the pathogenesis of periodontitis^4^. Hence, targeting pro-inflammatory mediators may be one of the most effective strategies for treating periodontitis. Previous reports indicate that reactive oxygen species (ROS) generation led to the development of osteoporosis by blocking the Nrf2/HO-1 signaling pathway. HO-1 is quite interesting as an antioxidant defence mechanism^5^. However, few studies have examined the influence of HO-1 on the differentiation of cells into osteoblasts. Periodontitis is characterized by alveolar bone loss, which depends on the fine balance between osteoclast-mediated bone resorption and osteoblast-mediated bone formation^6^. Moreover, bone resorption is influenced by the receptor activator of nuclear factor κ-B ligand (RANKL)^7^. This RANKL is necessary for osteoclast formation.

Lactic acid bacteria (LAB) are a popular probiotic present in most fermented foods worldwide. LAB showed anti-inflammatory, antioxidative, and immunoregulatory activities^8^. Thus, LAB is a safe and reliable functional food ingredient. Interestingly, some of the LAB strains are known to positively influence osteoblasts, osteoclast balance, bone inflammation, and bone resorption^9^. Similarly, *Ixeris dentata* (IXD) was demonstrated to have anti-inflammatory^10^, anti-proliferative^11^, neuroprotective^12^, and anti-allergic^13^ activities. Thus, the combination of the *Lactobacillus gasseri* (LG) strain and IXD potentially have a synergistic impact on reducing periodontal inflammation. This study aims to determine the effect of IXD/LGM anti-inflammatory activity and its associated bone differentiation on ligature-induced periodontitis in mice.

## Results

### *Ixeris dentata* (IXD) and *Lactobacillus gasseri* media (LGM) inhibit alveolar bone loss in a ligature-induced periodontitis model

**Figure 1:**
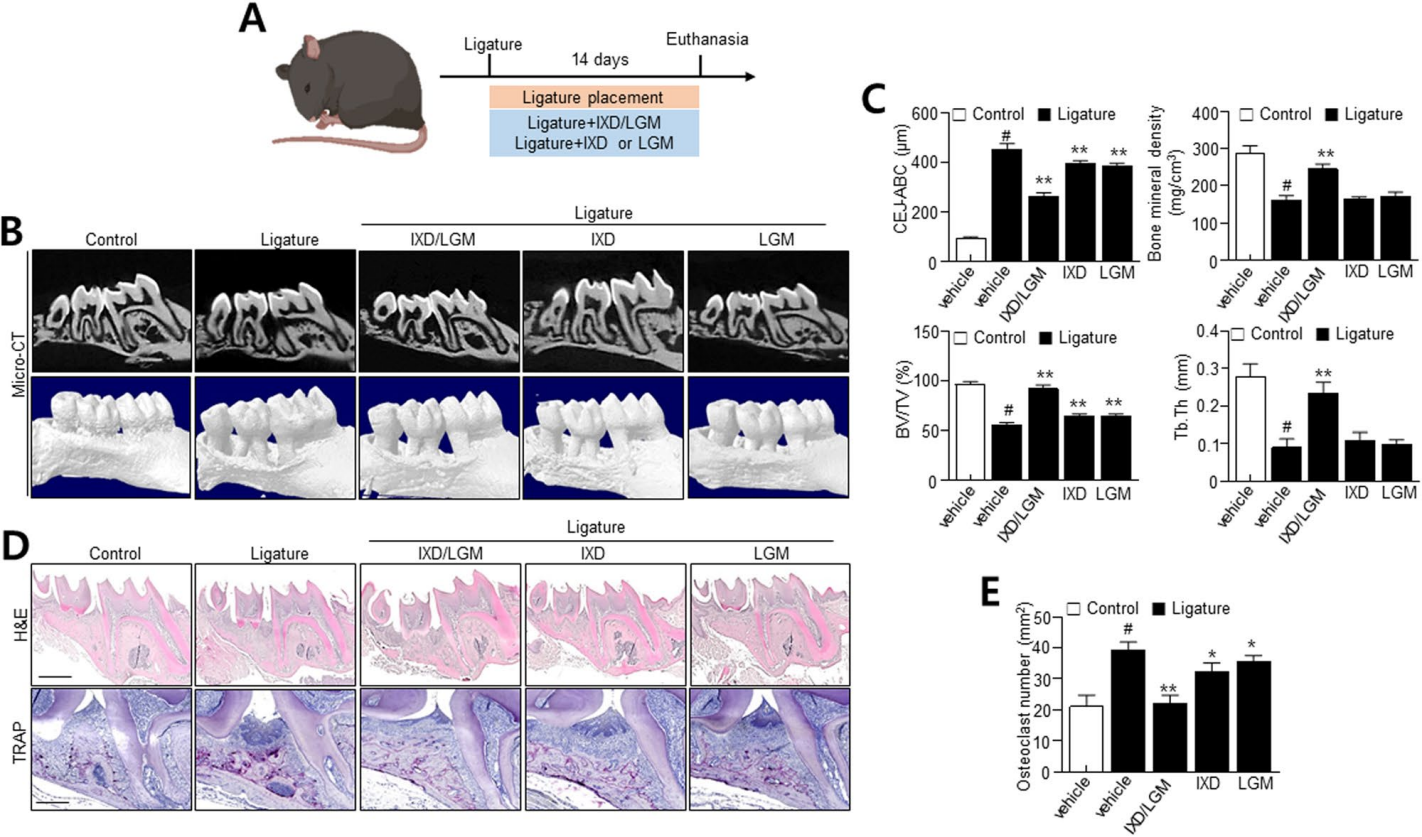
*Ixeris dentata* (IXD) and *Lactobacillus gasseri* media (LGM) extracts inhibit alveolar bone loss in ligature-induced periodontitis. (**A**) Experimental scheme. 7-week-old C57BL/6 J male mice were subjected to subcutaneous ligature insertion with or without IXD and LGM or co-treated IXD/LGM for 14 days. (**B**) Micro-CT (computed tomography) analysis of bone resorption state. (**C**) Quantification analysis of distance between cementum-enamel junction (CEJ) and alveolar bone crest (ABC), bone mineral density (BMD), BV/TV, and trabecular thickness (Tb. Th). (**D**) Histological analysis of the periodontium using hematoxylin and eosin (H&E) and TRAP staining. (**E**) Quantification analysis of osteoclast number. Each value represents the mean ± SEM. ^#^*p* < 0.05 vs. Control, *p < 0.05 vs. ligature, **p < 0.001 vs. ligature. Scale bars = 20 μm.

To confirm the efficacy of IXD and LGM against periodontitis, we applied the IXD/LGM to the ligature-induced periodontitis model. The upper left second molar was secured and held while the second molar was extracted. The socket of the extracted molar region was administered with the test compound for up to 2 weeks (Fig. 1A). The observations confirm that the loss of periodontal socket due to ligature was recovered with IXD, LGM, or IXD/LGM supplementation (Fig. 1B). Besides, micro-CT was used to determine the alveolar bone loss. This study observed alveolar bone loss between the first and second molars in ligatured mice. The single-treated IXD or LGM inhibited the alveolar bone loss, whereas co-treatment more significantly suppressed the alveolar bone loss than the single-treated group (Fig. 1C). Following ligature-induced periodontitis, there was 453.1 µm of alveolar bone loss. However, supplementation of 1 mg/mL IXD/LGM alveolar bone loss was limited to 393.86 and 386 µm, respectively. Consistently, the loss of bone mineral density (BMD) due to ligature-induced periodontitis was significantly restored by the single-treated IXD or LGM and IXD/LGM. Further, BMD markers like Bone volume/total volume (BV/TV) and trabecular thickness were recovered with IXD or LGM and IXD/LGM treatment. Comparatively, co-treatment with IXD/LGM resulted in a greater recovery effect against disruption. Additionally, BV/TV and Tb.Th was significantly reduced in the ligature group, while IXD or LGM and IXD/LGM co-treatment significantly enhanced the levels of BV/TV and Tb.Th. H&E staining observations indicate a protective effect by IXD or LGM and IXD/LGM (Fig. 1D). Next, TRAP^+^ cells confirm the osteoclast activity as indicated by osteoclastogenesis molecular markers. Here, IXD/LGM treatment significantly reduced the alveolar bone loss induced by the ligature placement and reduced the number of osteoclasts, indicating alveolar bone resorption (Fig. 1D,E). This further supports the results shown with respect to alveolar bone loss, where IXD/LGM significantly reduced the alveolar bone loss in the ligature-induced periodontitis model.

### *Ixeris dentata* (IXD) and *Lactobacillus gasseri* media (LGM) inhibit oxidative stress and pro-inflammatory cytokines in a ligature-induced periodontitis

**Figure 2:**
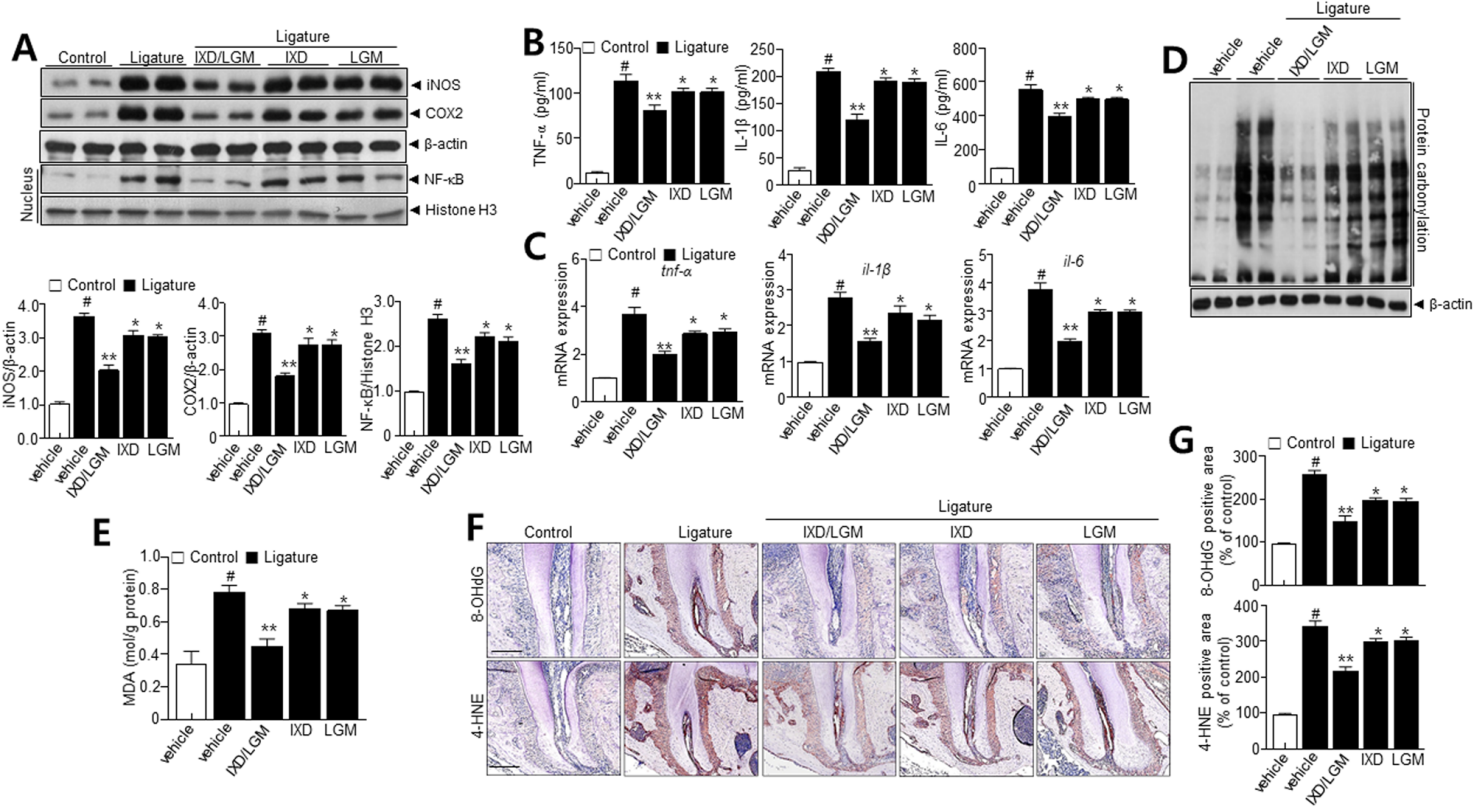
*Ixeris dentata* (IXD) and *Lactobacillus gasseri* media (LGM) extracts inhibit pro-inflammatory mediators and oxidative stress in ligature-induced periodontitis. C57BL/6 J mice were subjected to subcutaneous ligature insertion with or without IXD and LGM or co-treated IXD/LGM for 14 days. (**A**) Immunoblotting with anti-iNOS, COX-2, β-actin, NF-κB, and histone H3 antibodies and respective quantification analysis. (**B**,**C**) The expressions of pro-inflammatory cytokines and respective mRNAs. (**D**) Lysates from tissues analyzed for the presence of oxidized proteins by OxyBlot analysis. (**E**) Malondialdehyde (MDA) levels were analyzed as described in Materials and Methods. (**F**) Immunohistochemistry was performed with the 8-hydroxyguanosine (8-OHdG) and 4-hydroxy-2-nonenal (4-HNE) staining in tissues. (**G**) Quantification analysis was performed as described in the materials and methods. Each value represents the mean ± SEM. ^#^*p* < 0.05 vs. Control, *p < 0.05 vs. ligature, **p < 0.001 vs. ligature. The images of the original blots are available in Supplementary Information.

To investigate the anti-inflammatory effect of IXD and LGM in vivo model, we determined the effect of IXD, LGM, and IXD/LGM on the expression of pro-inflammatory cytokines and mediators. IXD/LGM significantly inhibited the expression of iNOS and COX-2 more than single-treatment with IXD or LGM (Fig. 2A). Also, NF-κB in the nucleus was increased in the ligature group, whereas IXD or LGM and IXD/LGM suppressed the nuclear factor-kappa B (NF-κB) in the nucleus. Consistently, IXD/LGM significantly inhibited the expression of pro-inflammatory cytokines like TNF-α, IL-6, and IL-1β (Fig. 2B). Additionally, IXD/LGM significantly suppressed the mRNA levels of these pro-inflammatory cytokines, confirming the influence of IXD/LGM (Fig. 2C). Together, these observations suggest the substantial impact of IXD/LGM in inhibiting inflammation in ligature-induced periodontitis. Oxidative stress plays a regulatory role in the progression of periodontitis^14^. Oxidative stress results in the modification of protein side chains to carbonyl derivatives (aldehydes and ketones)^15^. Carbonylation of protein disulfide isomerase (PDI) was enhanced in ligature-induced periodontitis and significantly reversed by IXD, LGM and IXD/LGM treatment (Fig. 2D). To investigate whether IXD/LGM protects oxidative stress against ligature-induced periodontitis, we performed malondialdehyde (MDA) assay. The results showed that ROS were accumulated in a ligature-induced model, and that treatment with IXD, LGM and IXD/LGM decreased the ROS accumulation (Fig. 2E). Additionally, we evaluated oxidative stress biomarkers, such as 8-OHdG and 4-HNE. Immunohistochemistry staining showed that rats in the ligature group showed significantly elevated levels of 8-OHdG and 4-HNE-positive cells, compared with those in control, while the 8-hydroxyguanosine (8-OHdG) and 4-hydroxy-2-nonenal (4-HNE) levels were reduced following IXD, LGM and IXD/LGM treatment (Fig. 2F,G). In the results, the IXD/LGM more significantly decreased the ROS and its associated oxidative signaling compared with either IXD or LGM.

### *Ixeris dentata* (IXD) and *Lactobacillus gasseri* media (LGM) extracts increase AKT-Nrf2-HO-1 signaling axis

**Figure 3:**
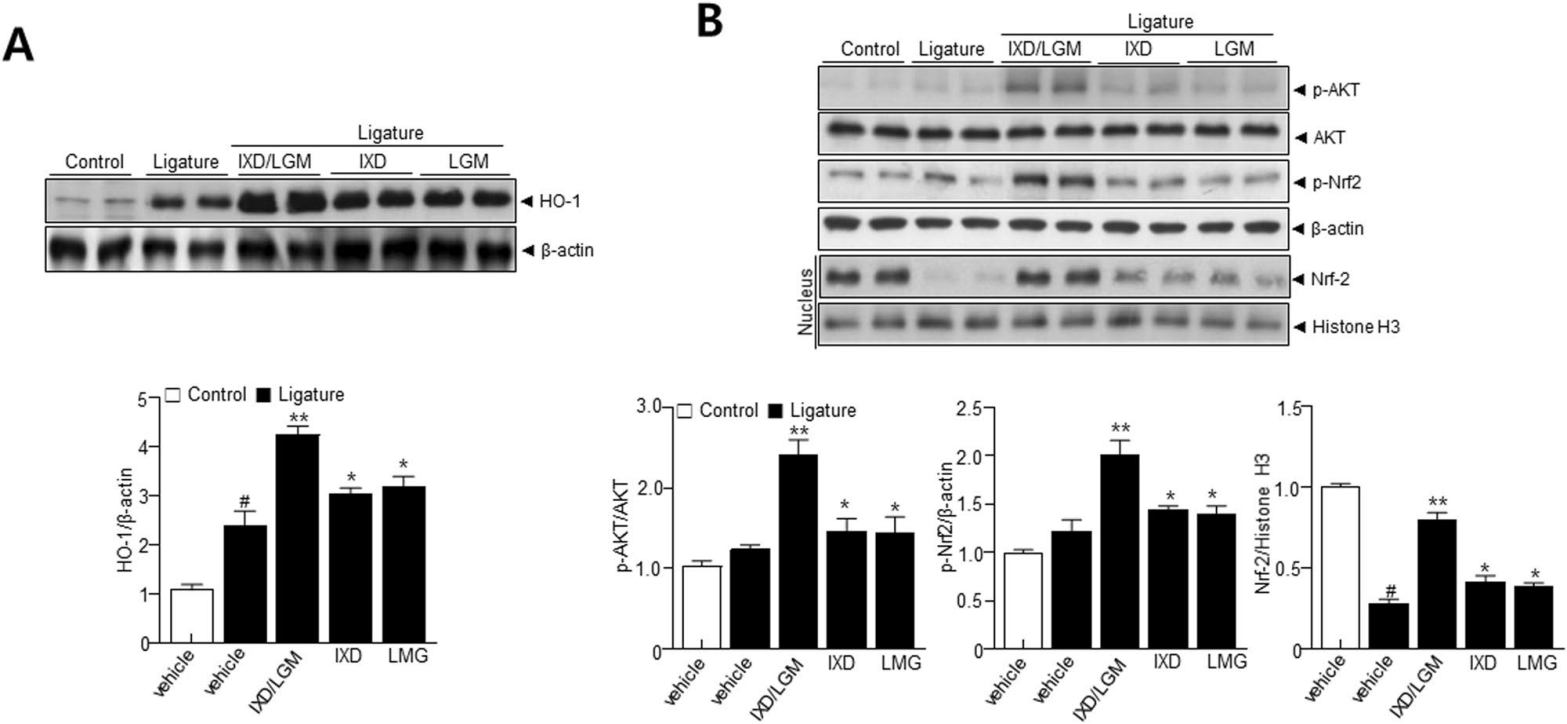
*Ixeris dentata* (IXD) and *Lactobacillus gasseri* media (LGM) extracts increase AKT-Nrf2-HO-1 signaling axis. C57BL/6 J mice were subjected to subcutaneous ligature insertion with or without IXD and *Lactobacillus gasseri* or co-treated IXD/LGM for 14 days. After isolating periodontium tissue, total lysis was performed as described in Materials and Methods. Immunoblotting was performed using anti-HO-1 and β-actin antibodies (**A**) and immunoblotting with anti-p-AKT, AKT, p-Nrf2, and β-actin antibodies (**B**). After isolating nucleus extracts, immunoblotting was performed using anti-Nrf-2 and histone H3 antibodies. Quantification analysis was performed as described in the materials and methods. Each value represents the mean ± SEM. ^#^*p* < 0.05 vs. Control, *p < 0.05 vs. ligature, **p < 0.001 vs. ligature. The images of the original blots are available in Supplementary Information.

AKT-Nrf2-HO-1 axis is considered as regulatory signaling against the ROS-based-inflammation axis. Thus, AKT-Nrf2-HO-1 axis was examined to determine the extent of influence of IXD or LGM and IXD/LGM in regulating periodontitis. The IXD/LGM significantly increased the expression of HO-1 more than the single-treated condition (Fig. 3A). Expectedly, the IXD/LGM increased phosphorylation of AKT compared with IXD or LGM (Fig. 3B). Nrf2, downstream hierarchy signaling, was more highly phosphorylated under the IXD/LGM than a single treatment and markedly translocated into the nucleus up on the IXD/LGM. These findings showed that IXD or LGM and IXD/LGM activated AKT-Nrf2-HO-1 axis and showed a synergistic effect of IXD and LGM on oxidative stress signaling.

### Osteogenic effect exerted by *Ixeris dentata* (IXD) and *Lactobacillus gasseri* media (LGM)

**Figure 4:**
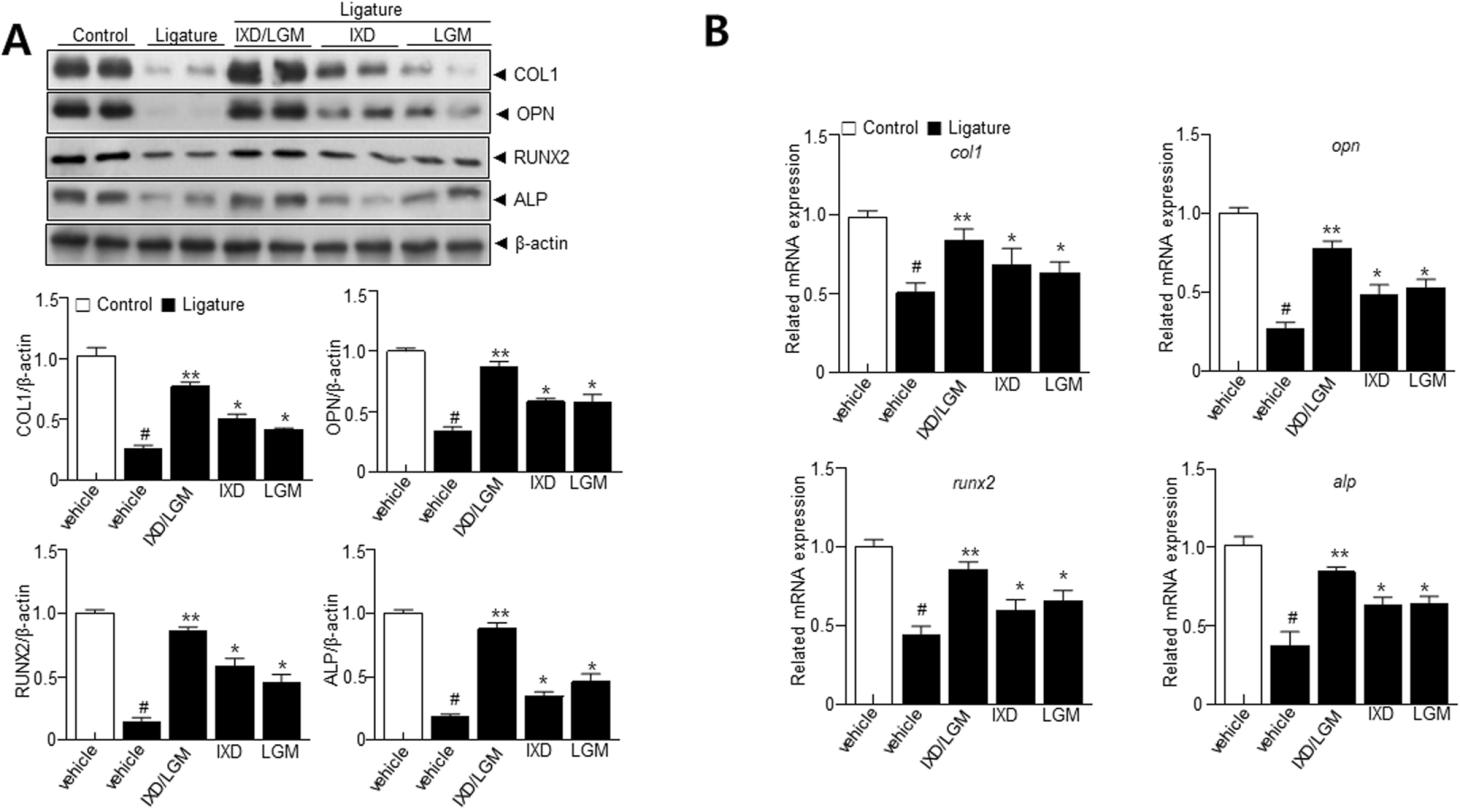
*Ixeris dentata* (IXD) and *Lactobacillus gasseri* media (LGM) extracts induce osteogenic differentiation in ligature-induced periodontitis. C57BL/6 J mice were subjected to subcutaneous ligature insertion with or without IXD and *Lactobacillus gasseri* or co-treated IXD/LGM for 14 days. (**A**) Immunoblotting using anti-COL1, OPN, RUNX2, ALP, and β-actin antibodies. (**B**) The mRNA levels of COL1, OPN, RUNX2, and ALP were measured by real-time PCR. The results were normalized with GAPDH. Each value represents the mean ± SEM. ^#^*p* < 0.05 vs. Control, *p < 0.05 vs. ligature, **p < 0.001 vs. ligature. The images of the original blots are available in Supplementary Information.

Reduced inflammatory response and recovery of alveolar bone loss are linked to osteogenic effects^16^. Hence, the influence of IXD and LGM extract on osteogenic differentiation was investigated. IXD or LGM and IXD/LGM significantly restored the expression of osteogenic proteins and mRNAs like collagen 1 (COL1), osteopontin (OPN), rant-related transcription factor 2 (RUNX2), and alkaline phosphatase (ALP) (Fig. 4A,B). Interestingly, IXD/LGM-induced inhibitory effect was significantly higher than IXD or LGM treatments.

### *Ixeris dentata* (IXD) and *Lactobacillus gasseri* media (LGM) mitigates LPS-induced oxidative stress via AKT-Nrf2-HO-1 signaling pathway in vitro

**Figure 5:**
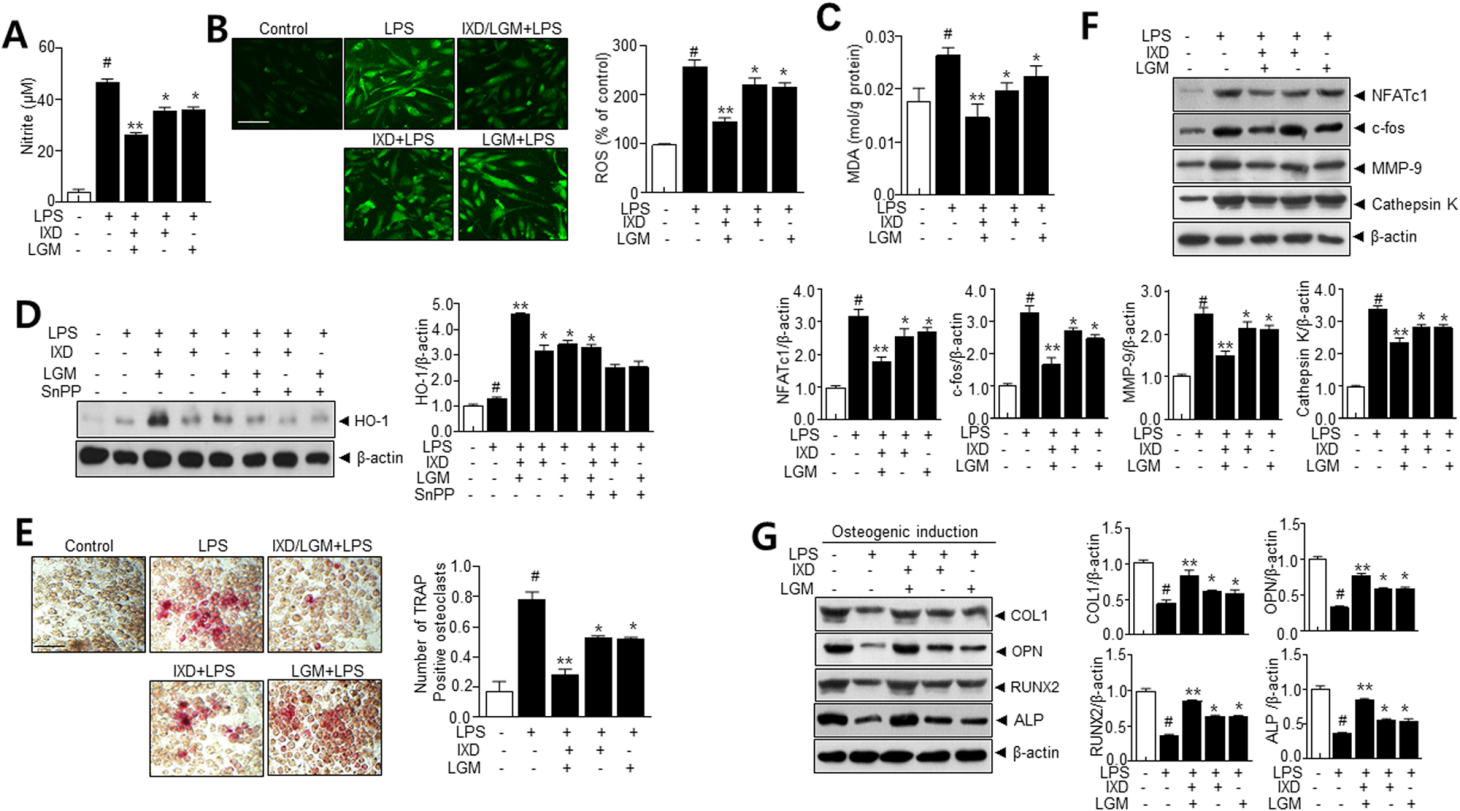
*Ixeris dentata* (IXD) and *Lactobacillus gasseri* media (LGM) extracts inhibit the RNAKL-induced osteoclast formation in RAW264.7 cells. RAW 264.7 cells were pretreated with IXD or LGM and the co-treated IXD/LGM in the presence or absence of SnPP (20 μM), a competitive inhibitor of HO-1, and then treated with LPS (1 μg/mL) for 24 days. The level of NO (**A**) and intracellular ROS measured by 2’, 7’-dichlorofluoresein diacetate (DCFH-DA) staining (**B**) (Scale bar = 200 μm) and quantification of DCFH-DA fluorescence intensity. (**C**) The MDA content of RAW 264.7 cells were measured by MDA assay kit. (**D**) Immunoblotting was performed with anti-HO-1 and β-actin antibodies. Quantification analysis was performed as described in the materials and methods. (**E**) Representative TRAP-stained images (Scale bar = 200 μm). Quantitative analysis of TRAP-positive cells. (**F**) Immunoblotting was performed using anti- NFATc1, c-fos, MMP-9, Cathepsin K, and β-actin antibodies. Quantification analysis was performed as described in the materials and methods. (**G**) Human dental pulp stem cells were pretreated with IXD or LGM and the co-treated IXD/LGM and incubated with 50 μg/mL ascorbic acid, 0.1 μM dexamethasone, and 10 mM β-glycerophosphate culture in osteo-induction medium for 7 days. Immunoblotting was performed using anti-COL1, OPN, RUNX2, ALP, and β-actin antibodies. Quantification analysis was performed as described in the materials and methods. Each value represents the mean ± SEM. ^#^*p* < 0.05 vs. Control, *p < 0.05 vs. LPS, **p < 0.001 vs. LPS. The images of the original blots are available in Supplementary Information.

To investigate whether IXD or LGM protects RAW 264.7 cells against LPS-induced oxidative stress, NO and ROS activity were measured. The NO and ROS was accumulated in LPS-treated RAW 264.7 cells, while IXD or LGM and IXD/LGM decreased the accumulation of NO and ROS (Fig. 5A–C). Additionally, HO-1 was measured under a similar condition where it was observed to be downregulated. IXD/LGM helped to recover HO-1 (Fig. 5D). To measure the effect of IXD or LGM on osteoclast formation in RAW 264.7, LPS (1 μg/mL) was used to induce TRAP-positive multinucleated osteoclast differentiation in RAW 264.7 cells. IXD and LGM extract showed inhibitory effects on TRAP-positive cells (Fig. 5E). Nuclear factor of activated T cells 1 (NFATc1) and c-fos are essential regulators to initiate osteoclast differentiation^17^. Thus, the NFATc1 and c-fos protein levels were determined to define the impact of IXD/LGM. Immunoblot observations confirm a significant decrease in NFATc1 and c-fos protein levels following IXD/LGM treatment (Fig. 5F). These results indicate that IXD/LGM potentially inhibits osteoclastogenesis. To investigate the protein levels of osteoblast markers in the early stages of osteoblast differentiation, we induced osteoblast differentiation for 7 days using α-MEM (minimum essential medium) containing β-glycerophosphate, ascorbate, and dexamethasone. The effect of IXD/LGM on osteoblast differentiation was evaluated by comparing the group treated with LPS alone with the group treated with LPS and IXD, LGM, or IXD/LGM. Osteogenic differentiation was significantly suppressed by LPS, which was recovered in the IXD or LGM and IXD/LGM (Fig. 5G).

## Discussion

The current study demonstrated that IXD and LGM inhibit inflammation, ROS accumulation, and progression of periodontitis. Specifically, the synergistic effect of IXD and LGM was also clarified throughout this study. At the molecular level, the Akt-Nrf2-HO-1 axis is suggested to be involved in the anti-inflammatory and differentiation effects in this ligature-induced periodontitis. Thus, IXD/LGM combination could be one of the alternative treatments for periodontitis.

The study showed that the IXD/LGM inhibits alveolar bone loss in a ligature-induced periodontitis model. The balance between osteoblasts and osteoclasts is essential to limit bone loss^18^. However, inflammatory environments created by periodontitis disrupt this delicate balance resulting in alveolar bone resorption and tooth loss^6^. Here, IXD/LGM inhibits alveolar bone loss in a ligature-induced periodontitis model by promoting alveolar bone resorption (Fig. 1B–D). Inflammation is the central process during bone resorption and must be managed to promote bone resorption. IXD/LGM inhibited TNF-α, IL-1β, and IL-6, which are crucial in the inflammatory responses (Fig. 2B,C). Also, IXD/LGM suppressed the pro-inflammatory mediator nitric oxide (NO). Interestingly, these investigations revealed that the synergistic effect of IXD and LGM is more effective than IXD or LGM (Figs. 1 and 2). These findings pave the way for IXD/LGM as an anti-inflammatory or functional food against alveolar bone resorption and inflammation in periodontitis.

Oxidative stress is crucial for regulating the course of periodontitis^14^. In this investigation, IXD/LGM was used as an antioxidant due to its proven antioxidant properties^19^ and anticipated protective effect against oxidative damage. Additionally, IXD contains polyphenols, which contribute to its ability to regulate free radicals. Specifically, luteolin 7-O-glucoside and luteolin 7-O-glucuronide are rich in IXD^10^. Periodontal pathogens stimulate an immune response elevating ROS production and inflammatory cytokine secretion^20,21^. Under these prolonged and persistent conditions, oxidative damage develops. Accordingly, an antioxidant-based therapy is required to prevent periodontitis under specific conditions. Here, IXD/LGM fulfills as an antioxidant-based therapy to regulate periodontitis by suppressing oxidative stress. Moreover, the study showed that the IXD/LGM enhances the Akt-Nrf2-HO-1 axis, an antioxidative and anti-inflammatory signal involved in the recovery of periodontitis. HO-1 activation is a common cellular response to oxidative stress; the increased expression and activity of HO-1 in Fig. 3 indicates the protective effect against oxidative stress. Further, Nrf2 acts along with antioxidant defence components to enhance cellular resistance to oxidative stress and suppress inflammatory responses by activating its target genes encoding HO-1^22^. Here, IXD/LGM significantly enhanced the expressions of HO-1 and p-Nrf2 in the ligature tissues of the periodontitis group compared with the ligature group (Fig. 3A,B). Consistently, HO-1 upregulation exhibited an anti-inflammatory effect via the production of pro-inflammatory cytokines and pro-inflammatory mediators, such as iNOS and COX-2^21^. Throughout this study, the HO-1-associated antioxidative signaling is suggested to control bone inflammation, which ultimately maintains the balance of bone remodeling process. Bone remodeling is composed of bone formation and resorption, which is based upon the balance between osteoblasts and osteoclasts. IXD/LGM has been shown to regulate oxidative stress-induced osteoclast differentiation (Figs. 2 and 5). Furthermore, COL, ALP, and OPN, the representative osteoblast differentiation markers, were increased by the IXD/LGM (Fig. 5), indicating the synergistic effect on the osteoblast and osteoclast balance leading to bone remodeling. The regulatory effect of these bone remodeling proteins defines the HO-1 signaling axis in vivo (Fig. 4A,B) and in vitro (Fig. 5F–G), suggesting the role of IXD/LGM in promoting bone remodeling process. In support, HO-1 has been identified as a potential bone regulatory effect in bone resorption/inflammatory periodontal disorders, including periodontitis^23^.

**Figure 6:**
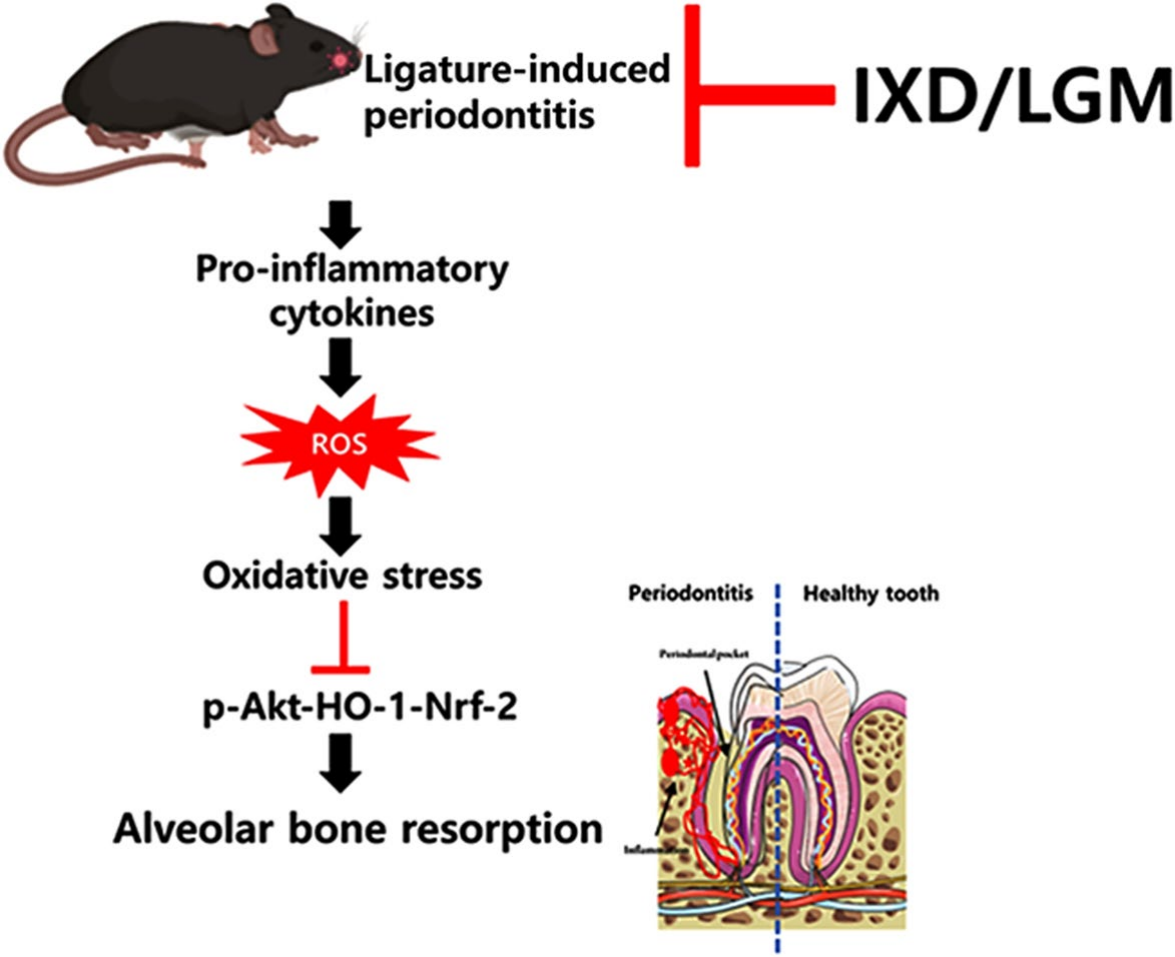
The proposed mechanism of *Ixeris dentata* (IXD) and *Lactobacillus gasseri* media (LGM) extracts against periodontitis effects.

In this study, the Akt-Nrf2-HO-1 signaling axis is disrupted in the inflammatory bone resorption, whereas the axis was significantly recovered in the IXD/LGM-treated condition. These observations collectively suggest the molecular mechanism involved in the protective effect of IXD/LGM against periodontitis (Fig. 6). In conclusion, IXD/LGM substantially protects against periodontitis by lowering oxidative stress and exerting an anti-inflammatory effect. Notably, the Akt-Nrf2-HO-1 signaling axis is involved in exerting this protective effect against periodontitis. Thus, IXD/LGM is a good candidate for therapeutic functional food against periodontitis.

## Materials and methods

### Chemicals and reagents

ELISA kits for TNF-α, IL-6, and IL-1β were procured from R&D system, Minneapolis, MN, USA.

The antibodies used in the study were as follows, β-actin (sc-47778), iNOS (sc-7271), COX-2 (sc-376861), NF-κB (sc-8414), HO-1 (sc-136960), p-AKT (sc-101629), ALP (sc-271431), COL1 (sc-59772), OPN (sc-21742), RUNX2 (sc-390351), NFATc1 (sc-17834), c-fos (sc-166940), MMP-9 (sc-393859), Cathepsin K (sc-48353), and Histone H3 (sc-517576) were purchased from Santa Cruz Biotechnology (Dallas, TX, USA). AKT (#9272), and Nrf2 (#12721) were purchased from cell signaling technology (Danvers, MA, USA). p-Nrf2 was purchased from Invitrogen (PA5-67520, Carlsbad, CA, USA). Polyvinylidene difluoride membrane (PVDF) and ECL-plus reagents (Healthcare Life Science, Tokyo, Japan). Bone morphogenetic protein-2 (BMP-2) from R&D Systems, Inc. and dexamethasone (Dex) from Sigma-Aldrich were obtained. Freshly extracted third molars from healthy patients were obtained from the Pusan National University Dental Hospital (Yongsan, Republic of Korea). Teeth were split under sterile conditions and pulp tissue was minced and plated in a 10 mm culture plate (Nunc, Roskilde, Denmark). Cells were cultured in StemMACS MSC Expansion Media (Miltenyi Biotec, Bergisch Gladbach, Germany) supplemented with human StemMACS MSC Expansion Media Kit XF (Miltenyi Biotec, Bergisch Gladbach, Germany), 10% fetal bovine serum (FBS, Gibco, Invitrogen), 100 U/mL penicillin and 100 mg/mL streptomycin (Gibco, Invitrogen) in a humidified atmosphere of 5% CO_2_ at 37 °C. For differentiation experiments, cells were cultured in osteogenic induction media with 50 μg/mL ascorbic acid (Sigma-Aldrich, St Louis, MO, USA) and 10 mmol/L β-glycerophosphate (Santa Cruz Biotechnology, Inc., Dallas, TX, USA).

### Ethical statement

This study was approved by the Animal Use and Care Committee of the Jeonbuk National University Hospital animal care and use committee (JBUH-IACUC-2022-12) conformed to the Guidelines for the Care and Use of Laboratory Animals based on the ARRIVE^24^. All methods were performed in accordance with the relevant guidelines and regulations.

### Animals and ligature-induced periodontitis model

Male C57BL/6J mice (Orient Science Co., Seongnam, Korea) aged 6 weeks (20–25 g) were housed in cages in 25 °C temperature-controlled chambers under a 12 h light/dark cycle (lights on at 08:00 h) with free access to food and water. A total of 62 mice were used in this study. All the animals were kept under standard living conditions with adequate food and water. After one week of acclimatisation, mice were randomly divided into 6 groups. Control group, orally administered with 100 μL phosphate-buffered saline (PBS); Ligature vehicle, administered with PBS; The IXD/LGM mixture was prepared by mixing 10^10^ CFU LGM and 10 mg/kg IXD in a ratio of 1:1. The co-treated conditions include the sequential treatment of 10 mg/kg/mice IXD and 10^10^ CFU/mice LGM, or the single-treated conditions include the 10 mg/kg/mice IXD and 10^10^ CFU/mice LGM. All the animals were administered orally twice per day. After 14 days of intervention, mice were euthanized to harvest maxilla with an intact palate and dissected to segregate left and right maxillae^25^. Harvested maxillae were autoclaved for 15 min. Next, defleshed maxillae were stained with 1% methylene blue (CAS: 7220-79-3, Sigma-Aldrich, St. Louis, MO, USA) for 1 min, followed by washing. Then 2D/3D-micro-CT was done to ascertain the bone structure and alveolar bone loss.

### Preparation of *Ixeris dentata* (IXD) and *Lactobacillus gasseri* media (LGM) extracts

The dry leaf of IXD was crushed and extracted with 70% ethanol at the ratio of 1.38% at 85 °C for 36 h. Then the filtrate was concentrated by using a rotary evaporator (EYELA, Japan) under vacuum. Lactobacillus strain MJM60645 was isolated from infant’s oral cavity as described in the previous study^26^. 16S rDNA sequence of MJM60645 showed 99% similarity with *Lactobacillus paragasseri*. *Lactobacillus gasseri* MJM6064 was isolated with human saliva and stored at − 80 °C with 20% glycerol until further use. The isolated MJM6064 strain was activated on a DeMan-Rogosa-Sharpe (MRS) agar plate and precultured for up to 16 h using MRS broth under standard culture conditions. Next, 500 µL preculture was scaled up in 0.5 L of MRS broth. Following fermentation, the supernatant was extracted with an equal volume of ethyl acetate (EtOAc). Later, the extract was dried using a rotary evaporator. The dried extract was suspended in distilled water for further applications.

### Cell culture

Murine macrophage cells (RAW 264.7) were purchased from American Type Culture Collection (ATCC, Manassas, VA, USA). RAW 264.7 cells were maintained in Dulbecco’s Modified Eagle Medium (DMEM) containing 10% FBS and 1% penicillin/streptomycin under standard conditions. RAW 264.7 cells were treated with LPS (1 μg/mL) for 24 days. For differentiation, the human dental pulp stem cells were cultured in α-MEM supplemented with 10% FBS and 1% penicillin/streptomycin (Gibco BRL, Grand Island, NY, USA), and cultured at 37 °C in a humidified atmosphere with 5% CO_2_. Human dental pulp stem cells were cultured at 1 × 10^4^ cells/well in a 6-well culture plate and then prepared for osteogenic induction, containing 50 μg/mL ascorbic acid, 0.1 μM dexamethasone, and 10 mM β-glycerophosphate culture in the osteo-induction medium for 7 days.

### Biochemical analysis

All the biochemical parameters and inflammatory cytokines were measured using the collected serum. Inflammatory cytokines TNF-α, IL-6, or IL-1β, were measured with commercial kits and performed as suggested by the manufacturer.

### Cytosolic and nuclear protein extraction

Collected tissue was minced on ice with radioimmunoprecipitation assay (RIPA) buffer. Tissue was homogenized with RIPA buffer (Thermo Fisher Scientific, Waltham, MA, USA), and noticeable fat junk was removed. A cytoplasmic extraction reagent kit (Pierce Biotechnology, Rockford, IL, USA) was used to separate cytoplasm and nuclei. Next, nuclei were washed and resuspended in a buffer. Later, sonicated and centrifuged to drive out debris. Protein contents were quantified using a Bio-Rad Protein assay buffer (Bio-Rad, Hercules, CA, USA).

### RT-PCR analysis

Total RNA was isolated with collected tissue using TRIzol/chloroform reagent (Bioneer, Daejeon, Korea). cDNA was synthesized with a PrimeScript-RT reagent kit (RR037A, Takara Bio Inc., Kusatsu, Japan) according to the manufacturer’s guidelines. qRT-PCR was carried out with PowerUp™ SYBR Green Master Mix (Thermo Fisher Scientific) by following manufacturer’s instructions. All primers used in qRT-PCR assay were listed as follows: tnf-α (F) 5′-GCCTCTTCTCCTTCCTGATCGT-3′, (R) 5′-TGAGGGTTTGCTACAACATGGG-3’; il-1β (F) 5′-AACCTCTTCGAGGCACAAGG-3′, (R) 5′- GTCCTGGAAGGAGCACTTCAT-3’; il-6 (F) 5′-AGTGAGGAACAAGCCAGAGC-3′, (R) 5′-GTCAGGGGTGGTTATTGCAT-3’; col1 (F) 5′-CCAGAAGAACTGGTACATCAGCAA-3′, (R) 5′- CGCCATACTCGAACTGGAATC-3’; opn (F) 5′—TCAGCTGGATGACCAGAGTG-3′, (R) 5′- TTGGGGTCTACAACCAGCAT-3’; runx2 (F) 5′- TCTTAGAACAAATTCTGCCCTTT-3′, (R) 5′-TGCTTTGGTCTTGAAATCACA-3’; alp (F) 5′—TGCAGTACGAGCTGAACAGG-3′, (R) 5′- GTCAATTCTGCCTCCTTCCA-3’; gapdh (F) 5′- TGTTCGTCATGGGTGTGAAC-3′, (R) 5′- GTCTTCTGGGTGGCAGTGAT-3’. Glyceraldehyde 3-phosphate dehydrogenase (GAPDH) was used as control, and the fold induction was estimated using the comparative 2^-ΔΔCt^ method and expressed as relative transcript levels.

### Immunoblotting

Immunoblotting was performed as outlined previously^27^. Briefly, cell lysates were separated by sodium dodecyl sulfate–polyacrylamide gel electrophoresis (SDS-PAGE), transferred onto a polyvinylidene difluoride (PVDF) membrane, blocked, and incubated overnight with the antibody as specified. All the protein signals were detected with ECL-plus reagents (Healthcare Life Science, Tokyo, Japan). Product intensity levels were analyzed with Image J (National Institutes of Health, Bethesda, MD, USA). All the values are normalized with values of β-actin.

### Protein carbonylation assay

Oxidative protein carbonylation assays were performed on tissue following western blot using an OxyBlot Protein Detection Kit (Millipore, Billerica, MA, USA) according to the manufacturer’s instructions^28^. The carbonyl groups in the protein side chains were derivatized to DNP-hydrazone by reaction with 2,4-dinitrophenylhydrazone (DNP-hydrazone) by reaction with 2,4-dinitrophenylhydrazine (DNPH) following the manufacturer’s instructions. After derivatization of the protein sample, one-dimensional electrophoresis was carried out on 10% electrophoresis gels. Proteins were transferred to PVDF membranes. After incubation with an anti-DNP antibody, the blot was developed using a chemiluminescence detection system.

### Nitrite assay

RAW 264.7 cells were pretreated with IXD or LGM followed by co-treatment with IXD/LGM for 6 h and then treated with LPS (Lipopolysaccharides from *Escherichia coli* O111:B4, 1 μg/mL) for 24 h. After the incubation, 100 μL of supernatant was added to 100 μL of Griess reagent (1% sulfanilamide, 2.5% phosphoric acid containing 0.1% naphthyl ethylene diamine). After 10 min of reaction, the absorbance was measured at 540 nm. At this time, sodium nitrite (NaNO_2_) was prepared for each concentration to show a standard curve, and the NO production amount was calculated.

### Histological staining

Collected periodontal tissue from the maxillary skull was fixed with 10% formalin (pH 7.5) overnight at 4 °C then transferred to a decalcifying solution with 0.5 M ethylenediaminetetraacetic acid-Na (EDTA-Na, pH 7.4) for 4 weeks. The EDTA solution was replaced weekly until the transparent surface of the bone tissue was visible. After decalcification was completed, the tissues were embedded in paraffin, and serial mesiodistal sections (5 μm) were stained with hematoxylin–eosin and analyzed microscopically (EVOS M5000 microscope, Invitrogen, CA, USA).

### TRAP staining

For tartrate-resistant acid phosphatase (TRAP) staining, the sections were stained using an acid phosphatase kit (PMC-AK04F, COSMO BIO USA, CA, USA). Deparaffinized sections were incubated with TRAP staining solution at 37 °C for 1 h until bright red TRAP was observed. After incubation, sections were rinsed three times in distilled water for 5 min and rinsed for additional five times with distilled water for 3 min. Next, sections were dehydrated quickly through graded alcohols, cleared in xylene, and mounted. The number of TRAP stain-positive osteoclast in the alveolar bone of maxillae was measured by histomorphometry, and calculated bone surface or bone area, the ratio of the osteoclast numbers/bone surface (N/mm^2^). The surface and area of bone in the alveolar bone of maxillae were measured by ImageJ software (NIH, Bethesda, MD, USA). The images were obtained using an optical EVOS M5000 microscope (Life Technologies, Carlsbad, CA, USA).

### Immunohistochemistry

The tissue sections were deparaffinized, removed xylene with ethanol, hydrated in graded alcohols, and placed in deionized water for 5 min. For immunostaining, antigen retrieval was done using 0.05% trypsin solution for 20 min at 37 °C and incubated the sections in 3% hydroperoxide for 10 min and incubated overnight with primary antibodies against 8-Hydroxy-2'-deoxyguanosine (8-OHdG 1:200, Abcam, Cambridge, MA) and 4-Hydroxynonenal (4-HNE, 1:200, Invitrogen, CA, USA) at 4 °C. After washing, the sections were incubated with the horseradish peroxidase (HRP)-conjugated secondary antibody. The HRP-conjugated antibody was visualized with a 3,3′-diaminobenzidine tetrahydrochloride (DAB) kit (DAKO, Carpinteria, CA). The sections were counterstained with hematoxylin. We counted the brown color spots as 8-OHdG and 4-HNE positivity using light microscope (EVOS M5000). For 8-OHdG and 4-HNE analysis, the images were converted to grayscale, and the threshold was adjusted for every image for background subtraction. The optical density in the slide was identified with ImageJ (NIH, Bethesda, MD, USA). All measurements were performed by the same individual who was blinded to the experimental conditions. The control group was assigned a mean value of 100, and changes are expressed relative to this value.

### Micro-CT (μCT) imaging and analysis

The fixed maxillae were subjected to Micro-CT scanning (Skyscan1275, Bruker-microCT, Kontich, Belgium) using a voxel size of 20 μm3 and a 0.5 mm aluminium filter at 55 kVp and 145 μA. To form a three-dimensional reconstruction, two-dimensional slices from each maxilla were combined using NRecon and CTAn/CTVol programs (Bruker, Kontich, Belgium). Bone loss was quantified by measuring the distance from the palatal and mesiobuccal cement-enamel junction (CEJ) to the alveolar bone crest (ABC). Bone mineral density (BMD) was calculated based on the thresholds within the raw data obtained from Micro-CT and the change in the direction of the coronal section. The region of interest was preset with the help of the interpolation method of the alveolar bone. Additionally, each sample was analyzed for trabecular thickness (Tb.Th) and bone volume to tissue volume (BV/TV).

### DCFH-DA staining

Intracellular ROS were assessed using 2′, 7′-Dichlorofluorescin Diacetate (H_2_DCFDA, Thermo Fisher Scientific, Waltham, MA, USA) staining. After staining with 10 μmol/L DCFH-DA at 37 °C for 30 min, the cells were collected and washed three times with PBS. Green fluorescence was examined under a fluorescence microscope with FITC filter (EVOS M5000 microscope (Life Technologies, Carlsbad, CA, USA). Cellular DCF fluorescence was subsequently quantified based on total pixel intensity of the images following conversion to 8-bit grayscale (ImageJ software; National Institutes of Health, Bethesda, MD, USA). The control group was assigned a mean value of 100, and changes are expressed relative to this value.

### Determination of lipid peroxidation activity

MDA was measured according to the method described^29^. The reaction mixture has TCA-TBA-HCl reagent and plant extract (50–250 mg/mL). The reaction mixture is heated to 90 °C for 10 min in a water bath, cooled and centrifuged at 10,000 rpm for 10 min to eliminate the pink-colored TCA precipitate. Ascorbic acid served as a standard. The amount of MDA produced by each sample was determined by comparing the absorbance of clear supernatant at 532 nm to a blank standard. The MDA levels are presented as mol/g protein.

### Statistical analysis

All the data were analyzed using GraphPad Prism v5.02 (San Diego, CA, USA). One-way ANOVA with Tukey’s post-hoc test was done for multiple comparisons. Data are expressed as the mean ± SEM. *p* < 0.05 indicate the statistical significance. For in vitro experiments, all immunoblots were performed in triplicate.

### Supplementary Information

Supplementary Figures.

## Data Availability

The datasets used and/or analyzed during the current study available from the corresponding author on reasonable request.
