# Supplementary figures and images for: Ixeris dentata and Lactobacillus gasseri media protect against periodontitis through Nrf2-HO-1 signalling pathway
Source: Sci Rep. 2023 Aug 8;13:12861. doi: 10.1038/s41598-023-39853-5 (PMC10409819; doi:10.1038/s41598-023-39853-5)

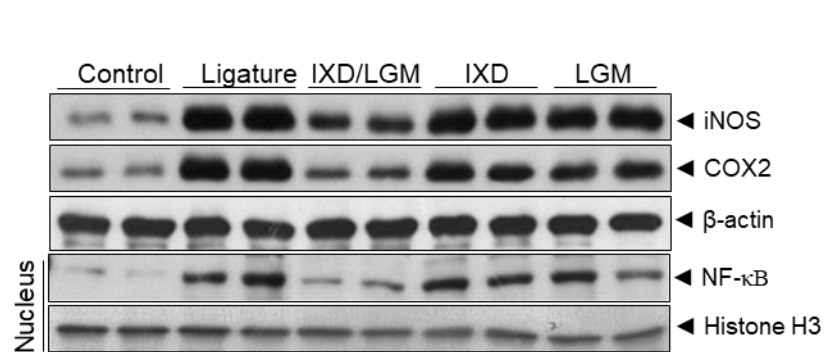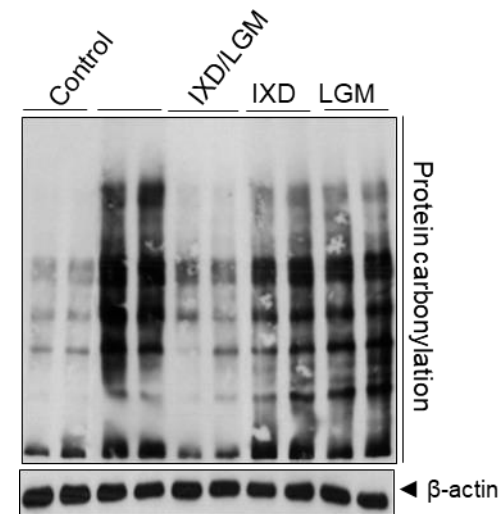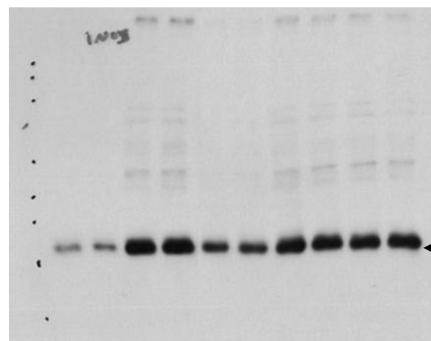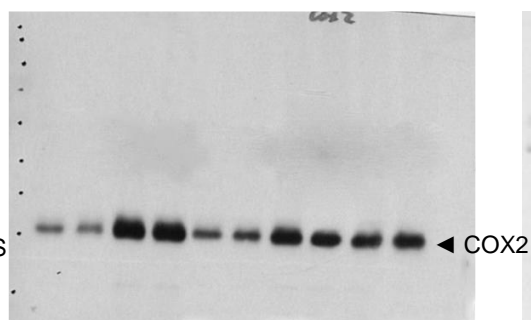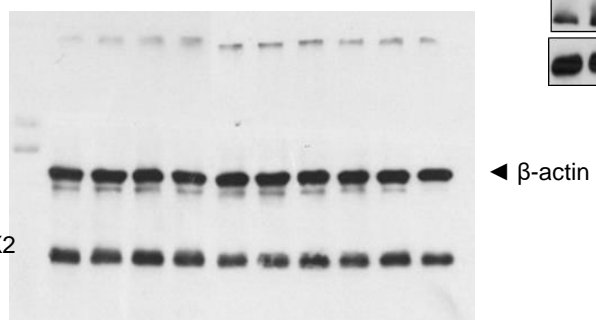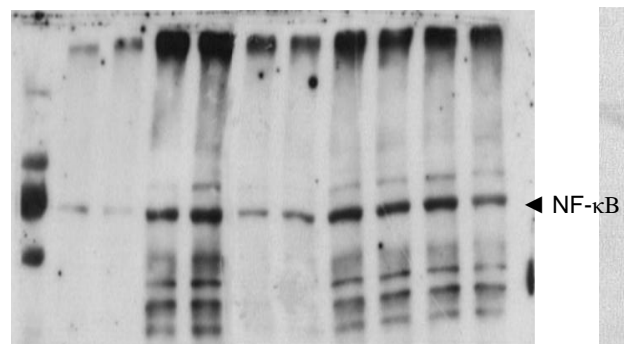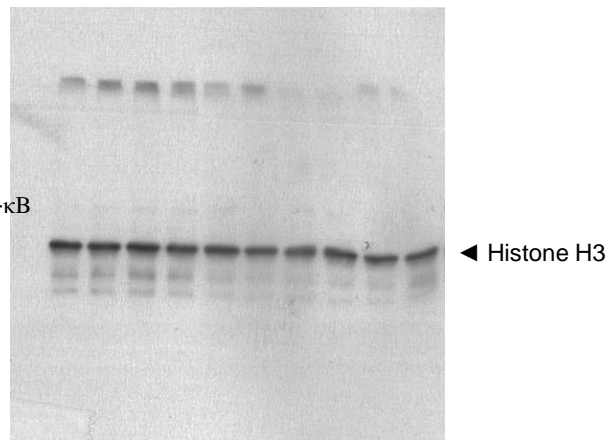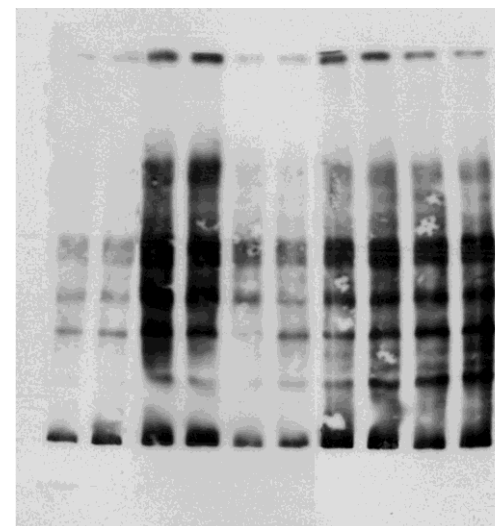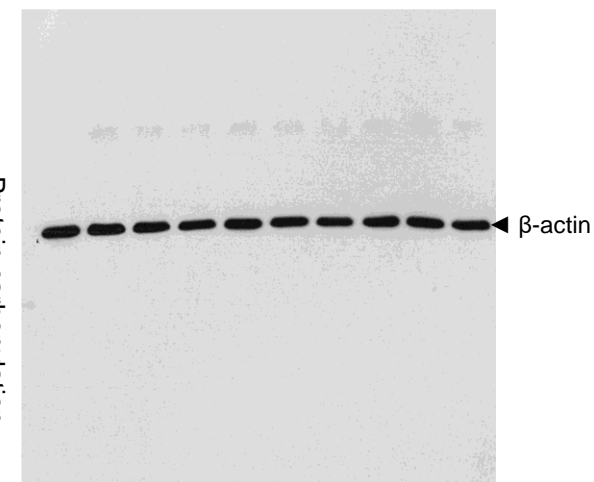

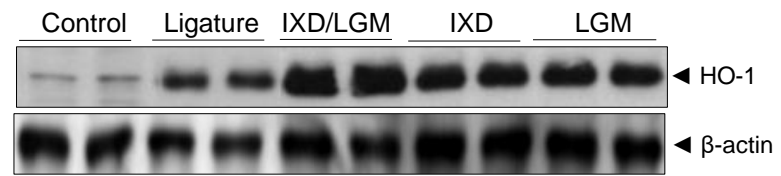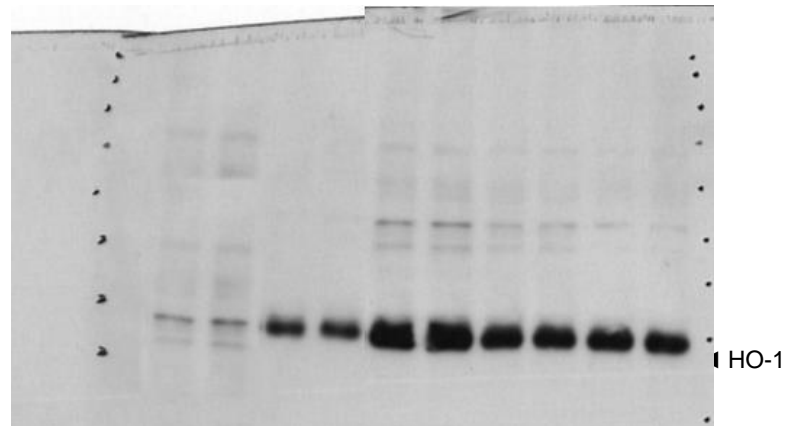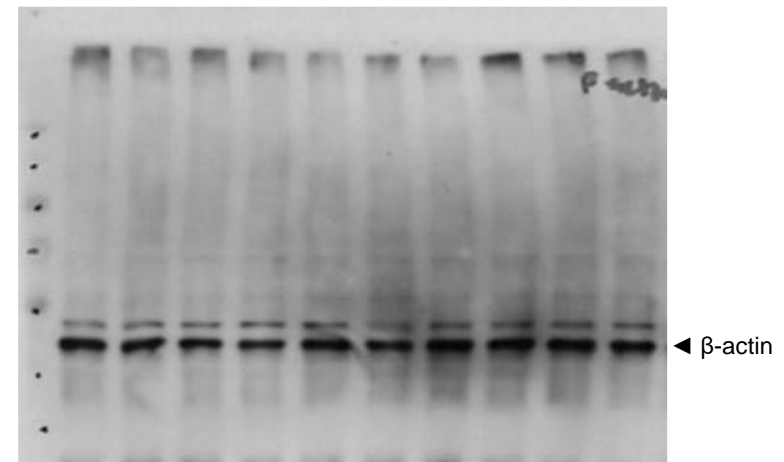

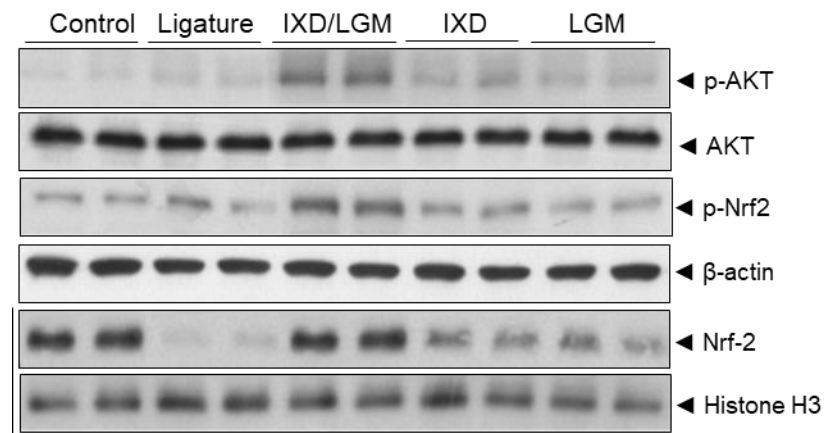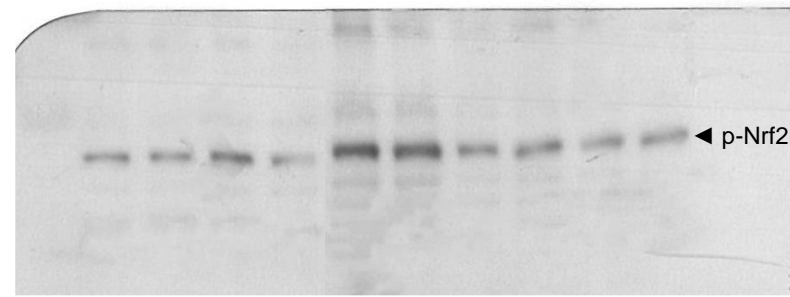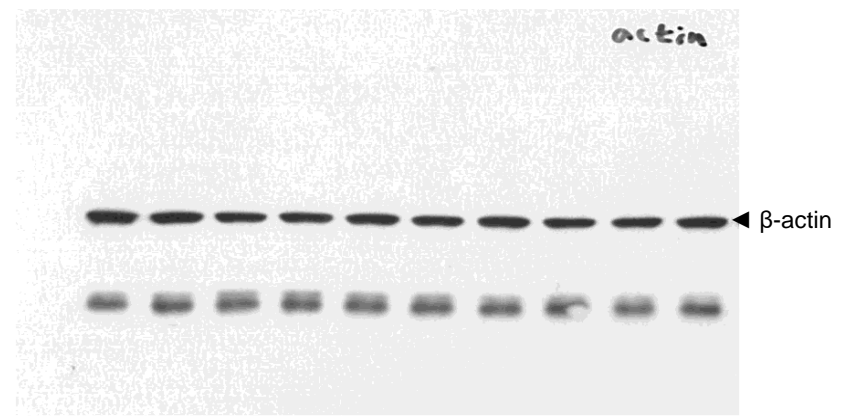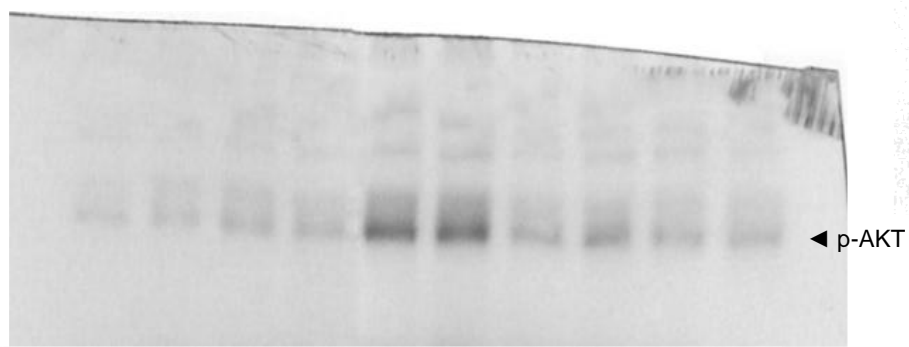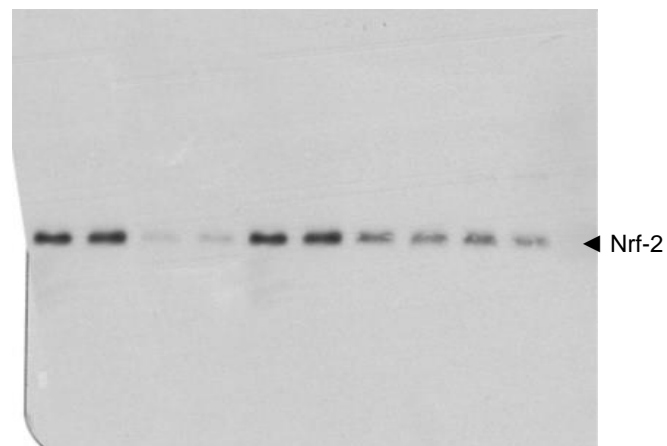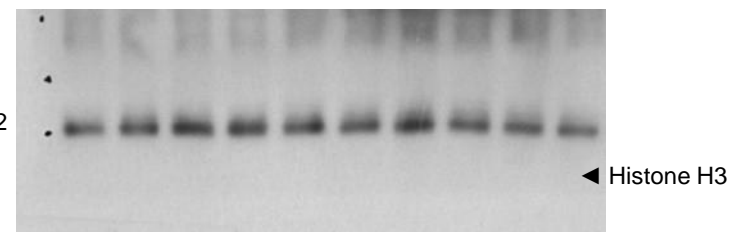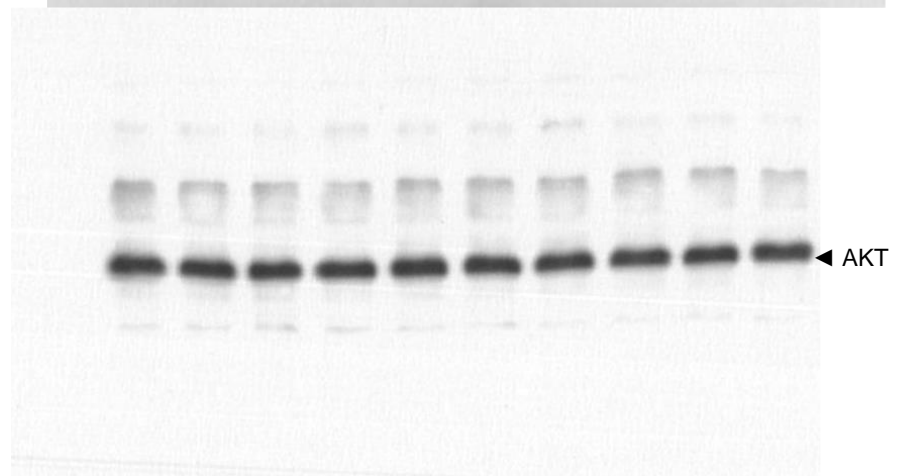

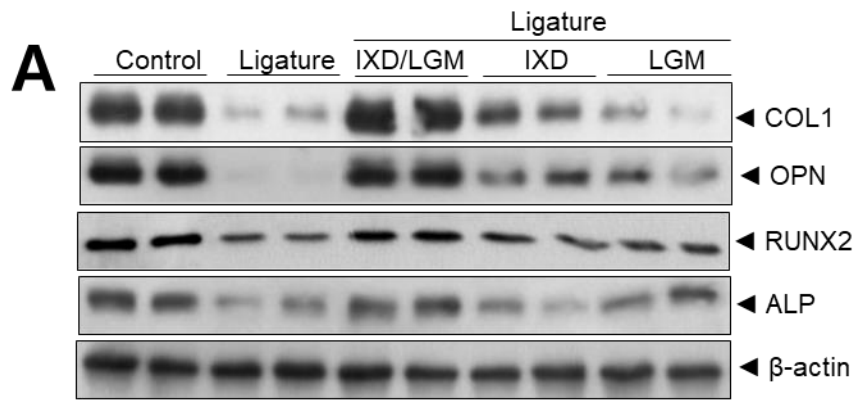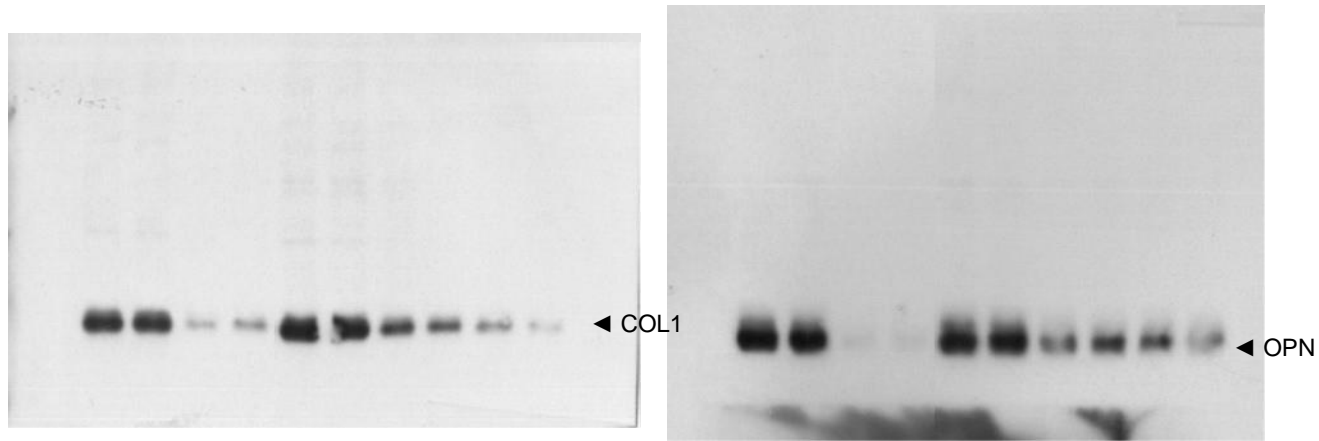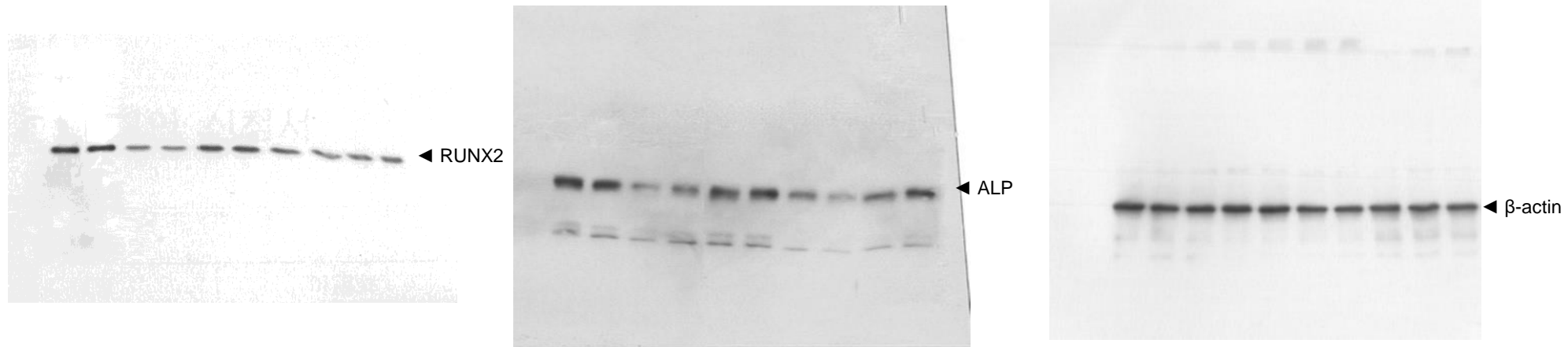

|      |   |   |   |   |   |   |   |   |
|------|---|---|---|---|---|---|---|---|
| LPS  | - | + | + | + | + | + | + | + |
| IXD  | - | - | + | + | - | + | + | - |
| LGM  | - | - | + | - | + | + | - | + |
| SnPP | - | - | - | - | - | + | + | + |

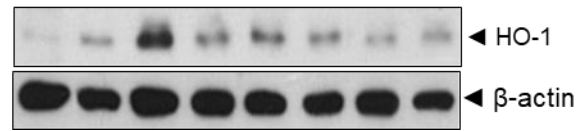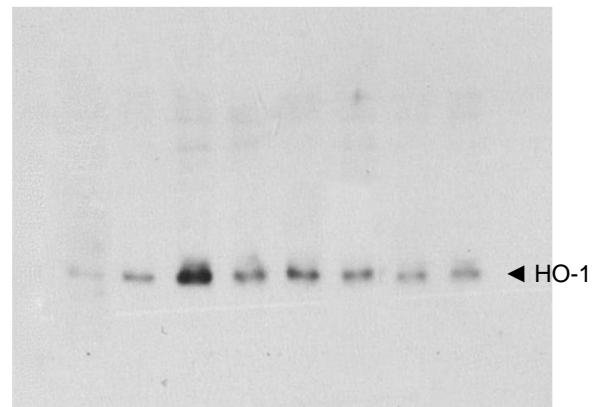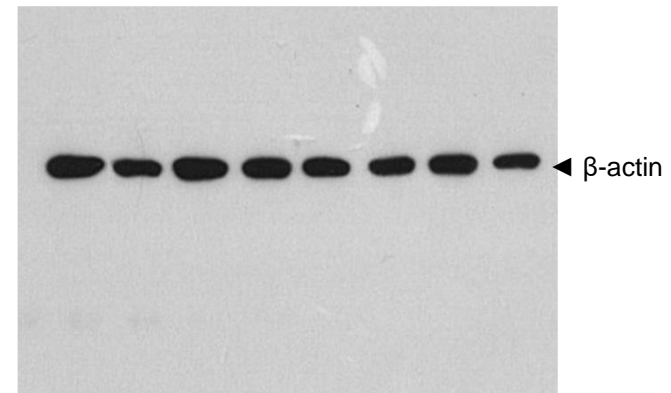

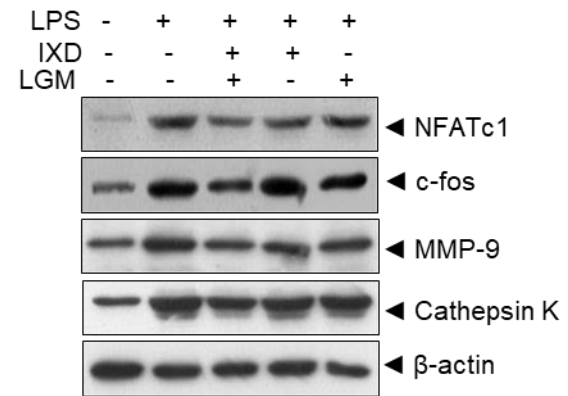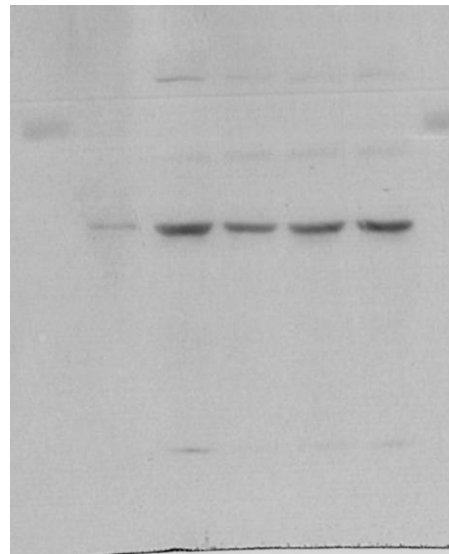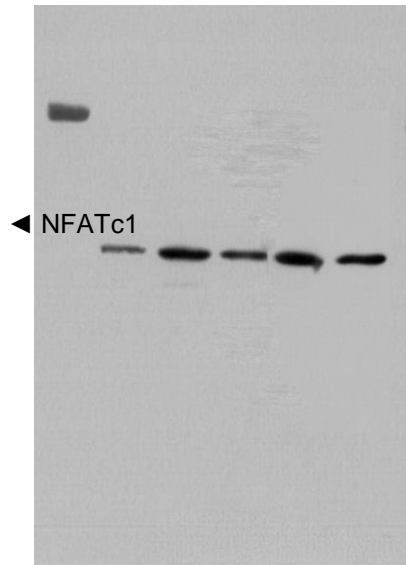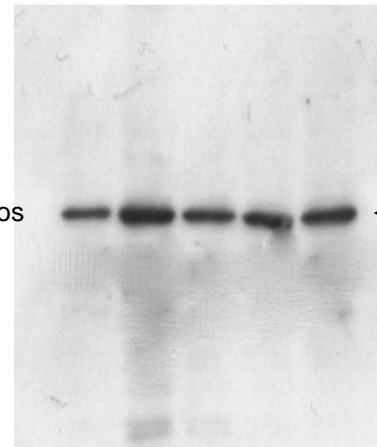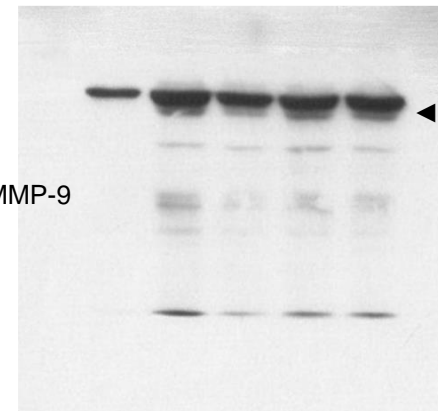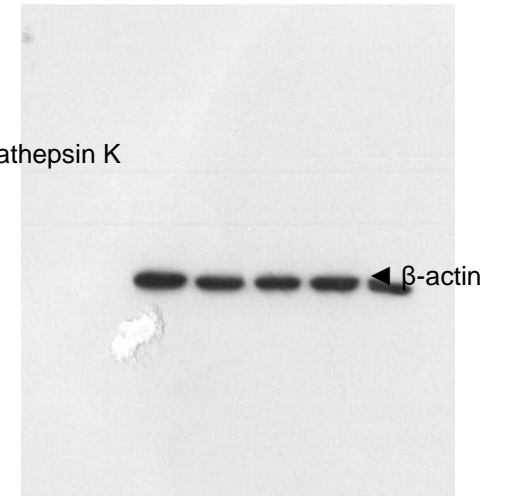

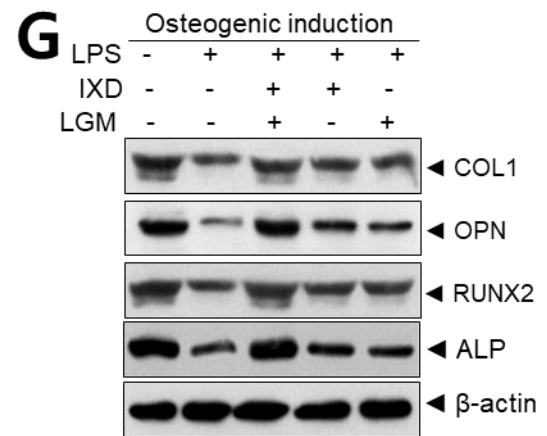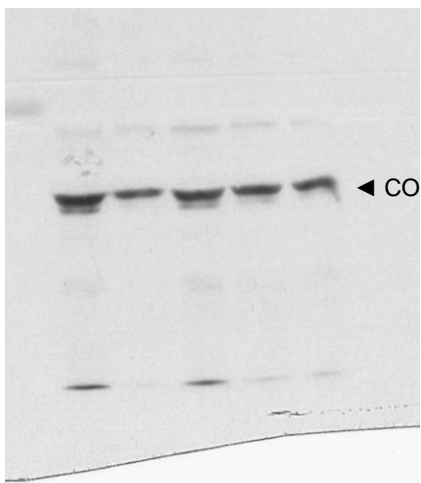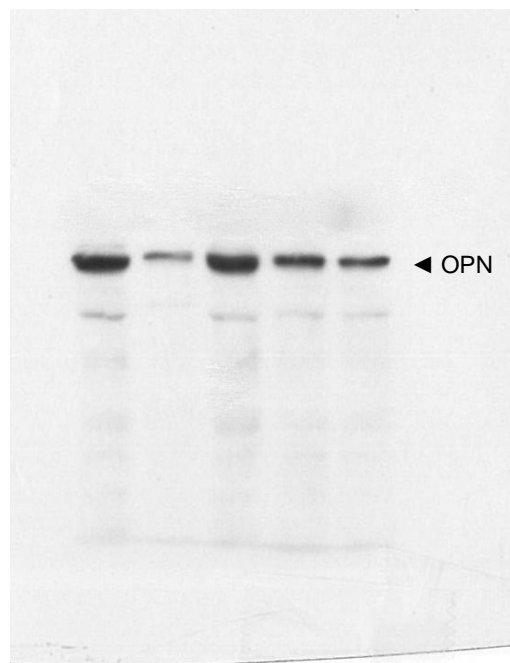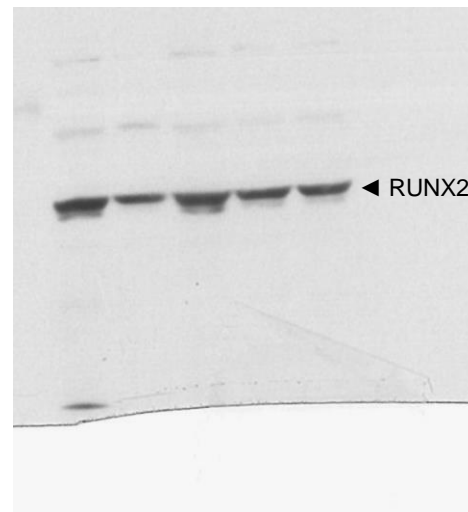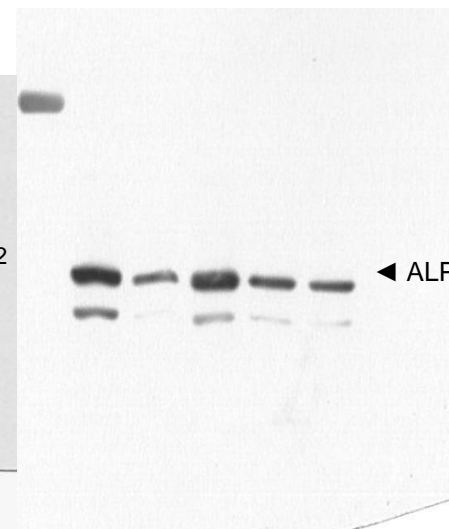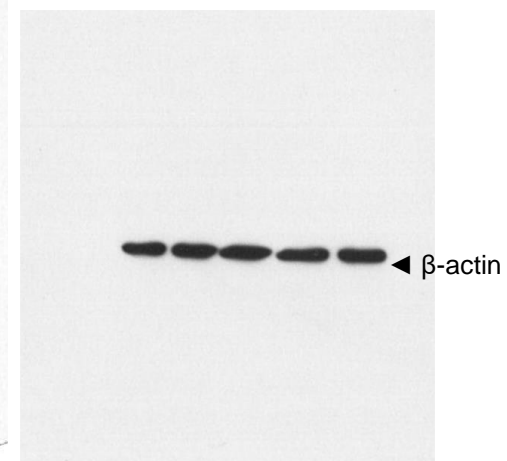

Supplement: Supplementary file 1 — Supplementary Figures. [file 41598_2023_39853_MOESM1_ESM.pdf]
